# Dietary Antioxidants: Micronutrients and Antinutrients in Physiology and Pathology

**DOI:** 10.3390/antiox8120642

**Published:** 2019-12-13

**Authors:** Ilaria Peluso

**Affiliations:** Council for Agricultural Research and Economics (CREA-AN), Research Centre for Food and Nutrition, 00178 Rome, Italy; i.peluso@tiscali.it

This Special Issue aimed to clarify the distinction between micronutrients and antinutrients and their different roles in physiology and pathology, considering the European Food Safety Authority (EFSA) criteria for health claims.

Nutrients include protein, carbohydrate, fat, fiber, sodium, as well as vitamins and minerals, whereas “other substance” means a substance other than a nutrient that has a nutritional or physiological effect [1]. Dietary antioxidants, with both antioxidant and pro-oxidant effects [2,3], include vitamins (tocopherol and ascorbic acid and provitamin-A carotenoids; essential micronutrients with physiological roles and symptoms of deficiency) and non-essential phytochemicals, including polyphenols. Godos et al. [4], in a meta-analysis, reported that dietary intake of anthocyanins (contained in pomegranate juice and berries) was associated with 8% reduction in risk of hypertension, when comparing highest versus lowest exposure, whereas intakes of total flavonoids or other subclasses showed a non-significant association. In addition to the suggested mechanisms for cyanidin 3-O-glucoside antihypertensive effect [4], cyanidin 3-O-glucoside was identified as one of the main hypoglycemic and hypolipidemic agents in wild blackberry extracts [5]. Cyanidin 3-O-glucoside showed a strong inhibitory activity on pancreatic lipase, higher than orlistat (commercial drug), and an acarbose-like effect [5]. These antinutrient effects could have pharmacological application for controlling hyperglycemia and hyperlipidemia. The EFSA Panel on Food Additives and Nutrient Sources added to Food (ANS) stated that applicants for claim should specify the intended target population (e.g., adults), the general population, or certain defined population subgroups [6]. In healthy subjects, the pharmacological inhibition of lipase was associated with an incremented excretion of fecal calprotectin and pro-oxidant activity of the colonic content [7].

The EFSA Panel stated that it should be clearly specified if certain subgroups of the population are excluded from the intended uses (e.g., pregnant and lactating women, infants) [6]. Rizzo et al. [8] concluded that evidence from clinical trials is inadequate to draft any definitive conclusion regarding vitamin D supplementation in pregnant women and there are still unanswered questions regarding vitamin D supplementation and target levels also for the general population [9,10].

Although, among celiac individuals, chocolate consumers had a higher lymphocyte-to-monocyte ratio [11] and a lower neutrophil-to-lymphocyte ratio [12], two hematological indexes of inflammation [13], while no differences were observed between chocolate consumers and non-consumers in red blood cell count, mean corpuscular volume, and hemoglobin values [11], a nutrition or health claim should not be made if it is inconsistent with generally accepted nutrition and health principles or if it encourages or condones excessive consumption of any food or disparages good dietary practice [1]. The COMMISSION REGULATION (EU) No 488/2014 (amending Regulation (EC) No 1881/2006) as regards maximum levels of cadmium in foodstuffs reported the following: “Chocolate and cocoa powder sold to the final consumer can contain high levels of cadmium and are an important source of human exposure. They are frequently consumed by children, e.g., chocolate as such or as sweetened cocoa powders used in cocoa beverages. When establishing maximum levels of cadmium, occurrence data for different types of chocolates and for cocoa powders sold to the final consumer should be considered. Since cadmium levels in cocoa products are related to their cocoa content, it is appropriate to establish different maximum levels of cadmium for products containing different percentages of cocoa. This should ensure that the maximum levels may also be complied with by chocolates with a higher percentage of cocoa.” [14]. Levels of flavanols comparable to those of formulated supplements can be reached with 180 g of apples or 8 g of dark chocolate, and a serving of 150–200 g of red fruits may yield amounts of flavanols and anthocyanins that are comparable to those of formulated supplements [15]. The EFSA Panel concluded that there is evidence from interventional clinical trials that the intake of doses equal to or above 800 mg epigallocatechin gallate (EGCG)/day taken as a food supplement has been shown to induce a statistically significant increase of serum transaminases in treated subjects compared to control [16]. Although the mean daily intake of EGCG resulting from the consumption of green tea infusions ranges from 90 to 300 mg/day, exposure by high-level consumers is estimated to be up to 866 mg EGCG/day [16]. Obesity stigma is an emergent phenomenon that can lead to significant negative health impacts [17], and liver toxicity has been reported in a 56-year-old woman who admitted that for obesity she had only been drinking broccoli juice (800 cc per day) during the preceding four weeks [18].

On the other hand, higher orthorexia nervosa risk has been reported in cancer patients [19]. Among polyphenols, curcumin, a dietary pigment and the main active component of the spice turmeric (*Curcuma longa*), has gained attention from scientists worldwide for its anticancer potential, but the results of human clinical trials have proven mostly to be inconclusive [20]. Toxicological data are included in the “Guidance on safety evaluation of sources of nutrients and bioavailability of nutrient from the sources” [6], and Cianfruglia et al. [21] studied the potential side effects of curcumin. 

In conclusion, as previously suggested by Calder [22], “nutrition science would be wise to adopt practices more akin to pharma when evaluating the functional properties and health impacts of foods, nutrients and non-nutrient food components”.
